# The Repression of Atoh1 by Neurogenin1 during Inner Ear Development

**DOI:** 10.3389/fnmol.2017.00321

**Published:** 2017-10-20

**Authors:** Héctor Gálvez, Juan J. Tena, Fernando Giraldez, Gina Abelló

**Affiliations:** ^1^DCEXS, Universitat Pompeu Fabra (UPF) - Parc de Recerca Biomèdica de Barcelona (PRBB), Barcelona, Spain; ^2^Centro Andaluz de Biología del Desarrollo, Consejo Superior de Investigaciones Científicas (CSIC), Sevilla, Spain

**Keywords:** neurogenesis, neural progenitors, cell fate, bHLH factors, Neurog1, sensory regeneration

## Abstract

Atonal homolog 1 (Atoh1) and Neurogenin1 (Neurog1) are basic Helix-Loop-Helix (bHLH) transcription factors crucial for the generation of hair cells (HCs) and neurons in the inner ear. Both genes are induced early in development, but the expression of Atoh1 is counteracted by Neurog1. As a result, HC development is prevented during neurogenesis. This work aimed at understanding the molecular basis of this interaction. Atoh1 regulation depends on a 3’Atoh1-enhancer that is the site for Atoh1 autoregulation. Reporter assays on chick embryos and P19 cells show that Neurog1 hampers the autoactivation of Atoh1, the effect being cell autonomous and independent on Notch activity. Assay for Transposase-Accessible Chromatin with high throughput sequencing (ATAC-Seq) analysis shows that the region B of the 3’Atoh1-enhancer is accessible during development and sufficient for both activation and repression. Neurog1 requires the regions flanking the class A E-box to show its repressor effect, however, it does not require binding to DNA for Atoh1 repression. This depends on the dimerization domains Helix-1 and Helix-2 and the reduction of Atoh1 protein levels. The results point towards the acceleration of Atoh1 mRNA degradation as the potential mechanism for the reduction of Atoh1 levels. Such a mechanism dissociates the prevention of Atoh1 expression in neurosensory progenitors from the unfolding of the neurogenic program.

## Introduction

Hair cells (HCs) of the inner ear are sensory mechanoreceptors that transduce hearing and balance stimuli into electrical signals. They are part of the functional unit of the inner ear, which is also composed of supporting cells (SCs) and neurons. In amniotes, multipotent neurosensory progenitors generate the three cell types with a tight time sequence. Neurons are specified first and delaminate from the otic epithelium and only later on in development, sensory precursors differentiate into HCs and SCs (Raft et al., 2007; Bell et al., 2008). Neuronal and HC development are driven by proneural type II basic Helix-Loop-Helix (bHLH) transcription factors Neurogenin1 (Neurog1) and Atonal homolog 1 (Atoh1), respectively (Ma et al., 1998; Bermingham et al., 1999; Alsina et al., 2004; Woods et al., 2004). Interestingly, the regulatory networks operating during development reactivate during HC regeneration. Birds are able to regenerate auditory HCs after damage and this ability relies on the competence of SCs to reactivate the expression of Atoh1 (Cafaro et al., 2007). In mammals, Atoh1 is also able to drive HC regeneration, but this ability is lost after early post-natal life (White et al., 2006; Lin et al., 2011; Kuo et al., 2015; Taura et al., 2016).

The expression of Atoh1 in HCs depends on several factors but it is ultimately established by an autoregulatory loop by which Atoh1 sustains its own expression (Helms et al., 2000). Both Atoh1 and Neurog1 are induced very early in development, but Atoh1 is repressed during neurogenesis and during the expansion of the prospective sensory organs (Puligilla et al., 2010; Neves et al., 2012; Abdolazimi et al., 2016). This, results in the delay of HC development with respect to neuronal production. The mutual exclusion between neurogenesis and sensorigenesis is paralleled by the antagonism between Neurog1 and Atoh1 (Matei et al., 2005; Raft et al., 2007). Yet, the molecular mechanisms underlying this interaction are largely unknown.

Atoh1 expression in the inner ear is accounted for by a 1.7 Kb enhancer region located 3.5 Kb downstream its coding region and composed of two different regions: Enhancer A and Enhancer B (Helms et al., 2000). There are several bHLH consensus binding sites, putative E-boxes CANNTG (Murre et al., 1994), in the Atoh1 promoter and in the 3’Atoh1-enhancer. The latter contains the essential site for Atoh1 autoactivation named Atoh1 E-Box Associated Motif (AtEAM), a 10-nucleotide palindrome with a class A E-box (E-box A) at its core, which is a genome-wide key binding motif for Atoh1 (Helms et al., 2000; Klisch et al., 2011). Yet, it is still unknown whether Neurog1 and other bHLH proteins interact with the 3’Atoh1-enhancer and if so, how this interaction occurs.

The goal of this work was to understand how Neurog1 counteracts the function of Atoh1. Reporter assay and Assay for Transposase-Accessible Chromatin with high throughput sequencing (ATAC-Seq) analysis show that enhancer B of the 3’Atoh1-enhancer is crucial for Atoh1 autoactivation and its repression by Neurog1. Both, activation and repression of the 3’Atoh1-enhancer are sensitive to the sequences flanking the E-box A in Enhancer B. However, Neurog1 does not require DNA binding to repress Atoh1 autoactivation and to interfere with HCs formation. Instead, the repressor function of Neurog1 depends on the H1-helix heterodimerization domain and results in the reduction of Atoh1 protein levels.

## Materials and Methods

### *In Ovo* Electroporation and Plasmids

HH12-14 (E2) chicken embryos (Granja Gibert, Spain) and HH20-21 (E3.5) otocysts were electroporated *in ovo* (Abelló et al., 2007; Kamaid et al., 2010). Reporters (1 μg/μl—Supplementary Table S1) and expression vectors (1–2 μg/μl) that are listed in Supplementary Tables S1, S2 were coelectroporated with EGFP-C1 (0.5 μg/μl, Promega) or pDsRed (0.5 μg/μl, Clontech) to select the electroplated cells and CMV-Luciferase (0.2 μg/ul) as internal control.

### β-galactosidase and Luciferase Reporter Assays

Protein extract from electroporated HH12-14 chicken otic vesicles was prepared using Reporter Lysis buffer (E397A, Promega) 24 h after electroporation (Petrovic et al., 2014). β-galactosidase and Luciferase measurements were done as in Neves et al. (2012). β-galactosidase activity was normalized for the level of electroporation using Luciferase reporter system.

### Site Directed Mutagenesis

**Figure 1:**
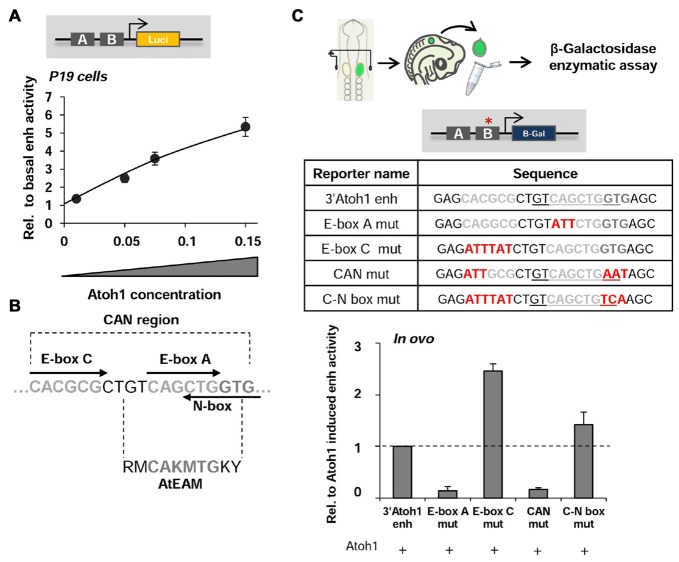
The 3’Atoh1-enhancer. **(A)** 3’Atoh1-enh activity depends on Atoh1 concentration. Quantification of the 3’Atoh1-enh reporter activity in the presence of increasing concentrations of Atoh1 in P19 cells. Values of luciferase activity (ordinates) are relative to those in the absence of Atoh1 (*n* = 5–7). **(B)** The CAN region of the 3’Atoh1-enh is the region of EnhB that contains an E-box A, surrounded by an E-box C and a reversed and overlapping N-box. **(C)** The activity of the 3’Atoh1-enh depends on E-box A and its flanking regions. The graph shows the values of β-galactosidase activity after co-electroporation of Atoh1 and the different constructs displayed in the table in E2 chicken embryos (*n* = 3–4). Mutations on E-box A and CAN abolished Atoh1-dependent activation, while mutation on E-box C caused the reverse effect. Mutation on E-box C and N-box without affecting the AtEAM shows no effect over Atoh1 activation (the two nucleotides following E-box A belonging to the AtEAM motif “GT” are replaced by “TC”). The red asterisk indicates the mutated region, the AtEAM sequence is underlined in the table for the 3’Atoh1-enh, CAN mut and C-N box mut.

Point mutations in the 3’Atoh1-enhancer reporter BgZA vector were introduced using the Quick Change XL site-directed mutagenesis kit (200517, Agilent Technologies). Detailed point mutations are shown in Figure 1C.

### Neurog1 Deletions and E-box Reporter Systems

Neurog1-∆H1 and Neurog1-∆H2 deletion expression plasmids were generated by inserting designed sequences (Ultramers 4 nmols, IDT) into a NcoI/XbaI pMiw digested plasmid. Neurog1-∆Cterm was generated by PCR using pMiw-mNeurog1 as a template. PCR product was digested with SalI/XbaI and inserted into Sall/Xbal digested pMiw. Sequences for IDT ultramers and PCR primers are available upon request.

4xEboxA and 4xCAN reporter constructs were generated by inserting annealed oligonucleotides (Ultramers 4 nmols, IDT) into a pGL3-basic vector (Promega) digested with NheI/BglII. The forward oligonucleotide contains a NheI site, while the reverse oligonucleotide contains a BglII site. 4xEboxA contains four copies of E-box A (4xTGT**CAGCTG**TCG), while 4xCAN contains four copies of the class C E-box (E-box C), E-box A and N-box (4xGAG**CACGCG**CTGT**CAGCTGGTG**AGC). The 4xEboxA multimer not affecting the AtEAM motif (Klisch et al., 2011) had the two nucleotides following the EboxA “GT” replaced by “TC”.

### P19 Transfection and Luciferase Reporter Assay

P19 cells (embryonic carcinoma cells derived from teratocarcinoma mice; Castro et al., 2006) were grown in DMEM with L-glutamine, antibiotics and 10% FBS. Ninety-six well dishes were seeded with 10^4^ cells and transfected at 24 h with Fugene HD (E2311, Promega), 75 pg of each expression plasmids, 75 pg luciferase reporter plasmid, and 30 pg pRenilla-TK vector (Promega) as internal control. Cells were lysed after 48 h and Luciferase/Renilla measurements were performed using Dual-Glo Luciferase Assay System (E2940, Promega). pGL3b-3’Atoh1-enh was generated by excising the 3’Atoh1-enhancer from 3’Atoh1-enh-BG-EGFP (J.Johnson’s Lab) with NheI/BglII and cloning it into a pGL3-basic vector (Promega) digested with NheI/BglII. The pGL3b-4xEnhB was cloned by digesting 4xEnhB-BgZA (Tg17, J-Johnson’s Lab) with BglII/SpeI and cloning into pGL3-basic vector (Promega) digested with NheI/BglII. CMVβ-hp300 (Eckner et al., 1994), pRc/RSV-mCBP (Richard Godman’s Lab), pCIG-hSmad1 (E. Martí, IRB, Barcelona) and the expression plasmids for *in ovo* electroporation (Supplementary Table S2).

### ATAC-Seq Analysis

ATAC-Seq experiments were performed as described in Buenrostro et al. (2015). Experiments on mice (C57BL/6, wild-type) were approved by the Ethics Committee of the Barcelona Biomedical Research Park (PRBB) and the Catalan Government. E10.5 mouse otocysts or E14 mouse cochleae were dissected, tissue was digested with Collagenase (C0130, Sigma, 0.3 mg/ml) during 45 min at 37°C and cells were suspended and counted in a Neubauer chamber. LY411575 treatment (University of Dundee, UK, 100 nM) was performed overnight on E14 mouse cochlea previous to ATAC-Seq procedure in a DMEM media with 1% FBS with L-glutamine and antibiotics. The resulting library was sequenced in Illumina Hi-seq 2000 pair-end lane. Reads were aligned with Bowtie2 Software (Langmead and Salzberg, 2012), using mouse July 2007 (mm9) as reference genome. Duplicated pairs or those ones separated by more than 2 Kb were removed. The enzyme cleavage site was determined as the position −4 (minus strand) or +5 (plus strand) from each read start, and this position was extended 5 bp in both directions (see Supplementary Figures S1, S2).

### Immunoprecipitation and Mass Spectrometry

2 × 10^6^ P19 cells were seeded and transfected with 4 μl PEI/μg DNA and 4 μg of pcDNA3.1-mAtoh1-FLAG (Quan et al., 2016) ± pMiw-mNgn1. Cells were lysed 48 h after transfection with buffer containing 1 mM EDTA, 100 μM Na_3_VO_4_, 0.5% Triton-X 100, 20 mM β-Glycerolphosphate, 0.2 mM PMSF in PBS with one complete EDTA free tablet (11873580001, Roche). Starting material for immunoprecipitation was 5–10 mg. The lysate was pre-cleared with Dynabeads protG beads (10003D, Thermo Fischer) during 2 h at 4°C, and incubated with 3–5 μg of 9E10, monoclonal mouse c-Myc (9E10, Santa Cruz Biotech) or rabbit FLAG (F7425, Sigma) overnight at 4°C. Dynabeads protG beads previously blocked (0.5% BSA) were added to the lysate and incubated for 2 h at 4°C. Beads were washed, suspended, digested and analyzed using a LTQ-Orbitrap Velos Pro mass spectrometer (Thermo Fisher Scientific, San Jose, CA, USA) coupled to a nano-LC (Proxeon, Odense, Denmark) equipped with a reversed-phase chromatography 2-cm C18 pre-column (Acclaim PepMap-100, Thermo; 100 μm i.d., 5 μm), and a reversed-phase chromatography 25 cm column packed with 1.9 μm C18 particles (Nikkyo Technos, Japan). SAINTexpress (Teo et al., 2014) was used to score protein interactions (see Supplementary Table S3).

### β-galactosidase Staining and Immuno -histochemistry

β-galactosidase staining and immunochemistry was done as in Neves et al. (2012). Primary antibodies were rabbit polyclonal neuronal class III β-tubulin (Covance PRB435P100; 1:500), rabbit polyclonal GFP (Torrey Pines 401, 1:400), mouse monoclonal GFP (Invitrogen A11120, 1:400), mouse monoclonal Myo7a (Hybridoma bank, 1:100), goat polyclonal Sox2 (Y-17, Santa Cruz, 1:400) and rabbit polyclonal DsRed (Takara, 632496, 1:400). Secondary antibodies were Alexa Fluor 488-, 555-, 546-, and 594-conjugated anti-mouse, anti-goat and anti-rabbit (Molecular Probes Invitrogen, 1:500).

### Immunoblot Analysis

Western blot was performed as in Neves et al. (2012). Protein samples were separated in 12% polyacrylamide gels, membranes incubated overnight with anti-FLAG (F7425, Sigma, 1:5000) or anti-GAPDH (Santa Cruz Biotechnology, Santa Cruz, CA, USA 1:15,000). Secondary antibodies goat anti-rabbit (P0488, Dako, 1:2000) and donkey anti-mouse (715-036-150, Jackson/Affinipure, 1:2000) coupled to horseradish peroxidase were incubated for 1 h at room temperature. Optical density of immunoreactive bands was quantified on a ChemiDoc XRS System and Quantity One Software v4.6.3 (Bio-Rad). Values were normalized to GAPDH.

### *In Vitro* Cultures of Otic Vesicles

*In vitro* cultures of otic vesicles were performed as described in Neves et al. (2011). Electroporated otic vesicles were incubated for 2–4 h with 10 μM MG-132 (M7449 SIGMA) for protein extraction or with 10 mg/mL ActinomycinD for mRNA assays (Petrovic et al., 2015).

### Statistics

Data are displayed as Mean ± SEM from at least three different experiments. *p*-values (t-Student’s test) were calculated and are at least below 0.05 for comparisons shown and discussed in the article. However, given the small size of the samples, and following Halsey et al. (2015), we show average values, SEM and the number of experiments, which give an estimation of effect size and precision.

## Results

### Neurog1 Repression of Atoh1 Autoregulatory Loop Occurs at the 3’Atoh1 Enhancer

The expression of Atoh1 during inner ear development relies on an enhancer located 3.5 Kb downstream its coding region, the 3’Atoh1-enhancer (Helms et al., 2000). Reporter activity of this enhancer is detected within the inner ear neurosensory domain (Neves et al., 2012). However, Atoh1 transcription is silent during neurogenesis and it is not expressed until later stages (Lumpkin et al., 2003; Neves et al., 2013). In the experiments that follow, we studied Atoh1 autoactivation and whether the 3’Atoh1-enhancer is regulated by Neurog1.

Reporter gene analysis of the 3’Atoh1-enhancer (herein the 3’Atoh1-enh) was carried out in chicken otic vesicles and in P19 cells. The 3’Atoh1-enh was activated by the overexpression of Atoh1 (Figure 1A). As shown by Helms et al. (2000), the E-box A (CAGCTG) in the AtEAM motif on Enhancer B mediates the autoactivation of Atoh1; and its mutation abolished the ability of Atoh1 to autoactivate the 3’Atoh1-enh reporter (Figure 1C). The AtEAM sequence is flanked by an E-box C (CACGNG) and by a reverse class N-box (CACNAG) that partially overlaps the E-box A (CANCTG; Figure 1B; Fisher et al., 1996; Iso et al., 2003). The region containing these three E-box binding sites is referred hereafter as the CAN region. Mutation of E-box C resulted in an increased activity of the reporter and, conversely, the combined mutation of E-box C, the AtEAM sequence and the N-box impaired the ability of Atoh1 to activate its own expression, mimicking the E-box A single mutation (compare E-box C mut and CAN mut in Figure 1C). The double mutation of class C and N-boxes (without affecting the AtEAM motif) neither prevented Atoh1 autoactivation nor activated the enhancer as the E-box C mutation alone did (compare E-box C mut and C-N box mut in Figure 1C). Together, the results suggest that the E-box C is important for 3’Atoh1-enh repression, while the integrity of the E-box A and the overlapping N-box are required for Atoh1 autoactivation.

**Figure 2:**
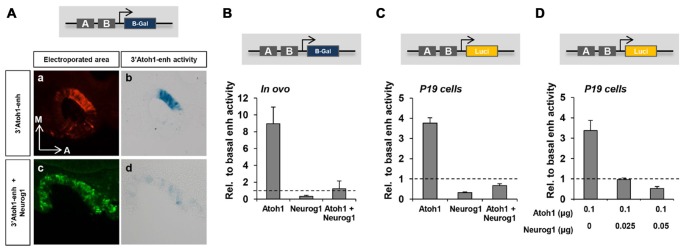
The repression of the 3’Atoh1 enhancer by Neurog1. **(A)** Neurog1 repressed the 3’Atoh1-enh in the otic vesicle. Otic vesicles were electroporated at E2 with the 3’Atoh1-enh w/o Neurog1. Otic vesicles were sectioned and processed for GFP immunoflurescence at E2+1 **(a,c)** or for β-gal reaction (**b,d**; *n* = 3). **(B)** Quantitation of 3’Atoh1-enh activity. Otic vesicles were isolated and β-gal activity measured in the three conditions indicated (*n* = 3–4). **(C)** Neurog1 repressed the activity of the 3’Atoh1-enh in P19 cells. Values of luciferase activity relative to the basal activity of the 3’Atoh1-enh in the conditions indicated in abscissa (*n* = 15). **(D)** Neurog1 is able to repress Atoh1 autoactivation at low concentrations in P19 cells. Values of luciferase activity corresponding to the 3’Atoh1-enh are represented against increasing concentrations of Neurog1 expressed as the ratio of electroporated Neurog1/Atoh1, concentrations ranging between 0 μg and 0.05 μg (*n* = 3–6).

We then studied whether Neurog1 is able to repress the activity of the 3’Atoh1-enh. In otic vesicles, the activity of the enhancer was restricted to the neurosensory domain (Figure 2Ab; Neves et al., 2012). Neurog1 abolished the activity of the 3’Atoh1-enh as shown by β-galactosidase *in ovo* staining and *in vitro* enzymatic quantification assays (Figures 2Aa–d,B compare values to the basal activity of the enhancer, dotted line). Moreover, Neurog1 was able to abolish the ability of Atoh1 to activate the 3’Atoh1-enh reporter (Figures 2B,C). Cotransfection of P19 cells with Atoh1 together with decreasing amounts of Neurog1 showed that the latter was able to prevent Atoh1 autoactivation even at a concentration ratio of 1:4 (Figure 2D).

### Dissecting the 3’ Atoh1 Enhancer: Enhancer B at the Core of Repression

**Figure 3:**
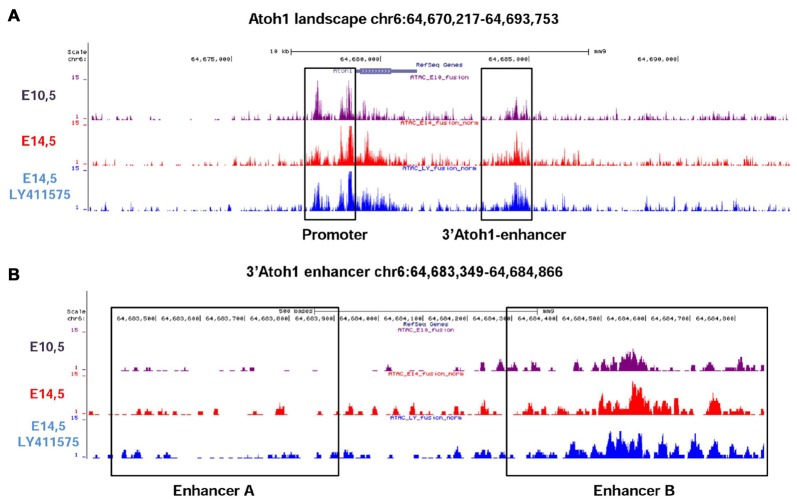
Assay for Transposase-Accessible Chromatin with high throughput sequencing (ATAC-Seq) analysis of Atoh1 gene landscape. **(A)** *In vivo* ATAC-Seq analysis of Atoh1 gene landscape. E10.5 mouse otic vesicles (upper lane) correspond to the neurosensory stage. Middle and lower lanes are from samples corresponding to E14.5 mouse cochleas either untreated (middle lane) or treated with the Notch inhibitor LY411575 (bottom lane). The regions mapping to the Atoh1 promoter and the 3’Atoh1-enh are boxed. **(B)** Enhancer B is the only accessible region of the 3’Atoh1-enh. In all conditions readings are mainly present at the position of EnhB, EnhA showing very little accessibility.

The 3’Atoh1-enh is divided into two different Enhancers, A and B. We used the ATAC-seq technique to analyze the accessibility of Atoh1 gene landscape *in vivo*. Mouse otocysts enriched in neurosensory tissue were analyzed at two different stages of development: E10.5 that corresponds to neurogenesis, when Neurog1 is expressed and Atoh1 is silent, and E14.5, when Atoh1 is expressed in differentiated HCs (Matei et al., 2005; Cotanche and Kaiser, 2010). A set of E14.5 cochleas were treated with LY411575, a gamma-secretase inhibitor that blocks Notch signaling (Ferjentsik et al., 2009). Under this condition bHLH Notch target genes Hes5/Hey1 are down-regulated, allowing Atoh1 expression and HC overproduction (Lin et al., 2011). The results show that the chromatin regions corresponding to the Atoh1 promoter and to the 3’Atoh1-enh were both accessible to transcription factor binding in all three conditions (Figure 3A; see Supplementary Figure S1 for ATAC-seq validation). However, zooming into the enhancer landscape showed that the region of Enhancer A was closed at all stages explored, suggesting that *in vivo* the activity of the 3’Atoh1-enh relies mainly on Enhancer B (Figure 3B).

**Figure 4:**
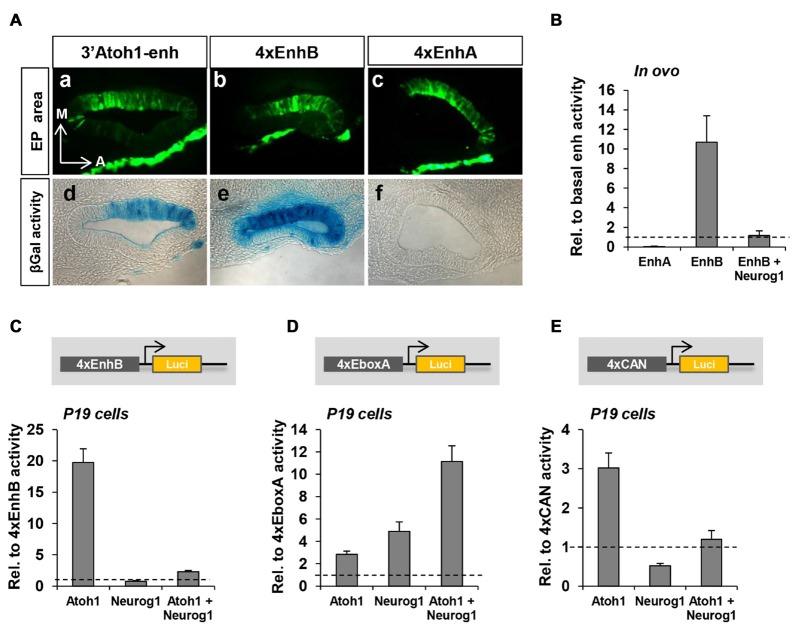
EnhB contains the essential elements for the repression by Neurog1. **(A)** EnhA shows no activity but regulates that of EnhB. Coronal sections of chicken otic vesicles (E2+1) electroporated with 3’Atoh1-enh **(a,d)**, 4xenhB **(b,e)** or 4xEnhA (**c,f**; *n* = 3). EP area = Electroporated area. **(B)** Enhancer activity quantitation in electroporated E2+1 otic vesicles in the three conditions indicated. Values are relative to the basal β-gal activity of the 3’Atoh1-enh. Isolated EnhA showed very low activity, whereas EnhB showed an increased activity that was suppressed by Neurog1 (*n* = 3). **(C)** Neurog1 counteracts Atoh1 induced activity of EnhB in P19 cells (*n* = 13). Luciferase activity corresponding to EnhB in the three conditions indicated in abscissa. **(D)** Atoh1 and Neurog1 activated the isolated E-box A. The activity of 4xE-box A is shown for the three conditions indicated in abscissa (*n* = 5–7). **(E)** Neurog1 turned into a repressor when E-box A was flanked by C and N-boxes (4xCAN multimer *n* = 5–6).

To further analyze the contribution of each enhancer to Atoh1 regulation, separate multimer constructs of either Enhancer A or B (4xEnhA or 4xEnhB) were electroporated and their activity measured in otic vesicles. The spatial activity of 4xEnh A and B was strikingly different in chick otic vesicle. 4xEnhA showed no activity at all (Figures 4Ac,f). In contrast, 4xEnhB exhibited a strong signal (Figures 4Ab,e) that expanded beyond the normotopic 3’Atoh1-enh activity domain (Figures 4Aa,d). Quantitation of reporter activity showed undetectable endogenous activity of 4xEnhA, and no activation by Atoh1 or Sox2 (data not shown). This contrasted with the intense activity of 4xEnhB (Figure 4B). These results correlate well with the different chromatin accessibility of Enh A and B revealed by ATAC-seq analysis. Next, we studied whether Enhancer B was also repressed by Neurog1. Indeed, 4xEnhB activity was strongly reduced by Neurog1 *in ovo* and in P19 cells, where Neurog1 also prevented Atoh1 autoactivation (Figures 4B,C). This indicates that Enhancer B accounts for the repression by Neurog1.

### Repression by Neurog1 Requires the CAN Region

We further focused on the CAN region of EnhB and explored the behavior of two multimer constructs: (a) E-box A multimer consisting of four tandem repeats of the E-box A without the two flanking E-boxes (tgt**CAGCTG***tc*g, 4xEboxA); and (b) CAN multimer consisting of four tandem repeats of E-box C, E-box A and the N-box (gag**CACGCG**CTGT**CAGCTGGT**Gagc, 4xCAN). Atoh1 and Neurog1 are both type II bHLH transcription factors that act as transcriptional activators preferentially binding to E-box A (Murre et al., 1994; Massari and Murre, 2000). In agreement, they activated the 4xEboxA multimer (Figure 4D), the mixture of both being additive. However, Neurog1 activation turned into repression when tested on the 4xCAN construct (Figure 4E).

### Neurog1 Does Not Require Direct Binding to DNA for Atoh1 Repression

From the above results, there may be three major and not mutually exclusive mechanisms for the repression of Atoh1 by Neurog1. First, the direct interaction of Neurog1 with the CAN region, secondly, the interaction Neurog1 with Atoh1protein or with essential cofactors for Atoh1 autoactivation and, finally, the post-transcriptional modification of Atoh1 at mRNA or protein levels.

**Figure 5:**
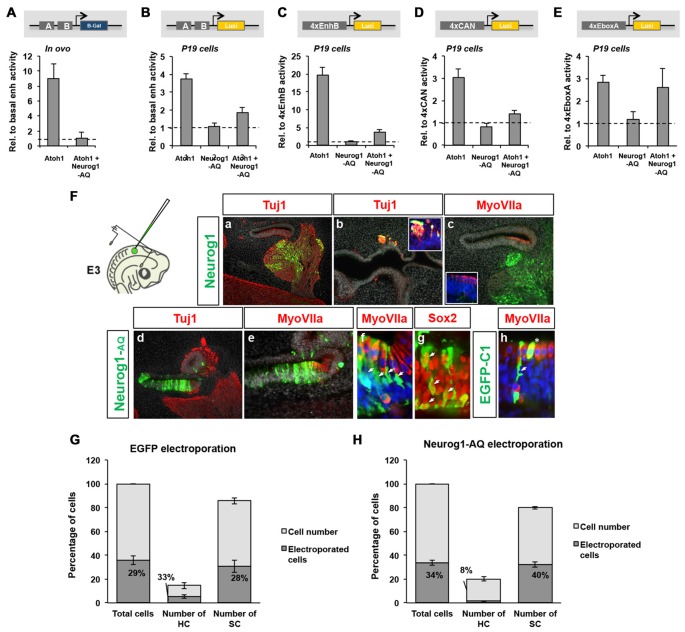
Neurog1 does not require DNA-binding to repress Atoh1. **(A)** Neurog1-AQ is able to repress Atoh1 dependent activation of the 3’Atoh1-enh *in vivo*. Quantitation of β-gal activity in otic vesicles after electroporation of Atoh1 alone, or with Neurog1-AQ. Values are relative to the basal β-gal activity of the 3’Atoh1-enh (*n* = 3). **(B)** Neurog1-AQ is able to repress 3’Atoh1-enh in P19 cells. Quantitation of luciferase activity in P19 cells in the conditions indicated (*n* = 5–15). **(C)** EnhB accounts for the repression by Neurog1-AQ (*n* = 4–13). **(D)** Neurog1-AQ is able to repress the CAN reporter. Experiment like in **(C,D)**, but after electroporation of the 4xCAN (*n* = 3–7). **(E)** Neurog1-AQ does not interfere with E-box A in isolation (*n* = 3–7). **(F)** Neurog1-AQ is able to prevent hair cell (HC) formation. Neurog1 (upper row) or Neurog1-AQ (lower row) were electroporated *in ovo* (E3.5) and otic vesicles examined for neuron (Tuj1), HC (MyoVIIa) or SC (Sox2) markers at E6.5. Cells electroporated with Neurog1 adopted neuronal fate (**a–c**, cvg: cochleo-vestibular ganglion), while those with Neurog1-AQ **(d–h)** turned into supporting cells (SCs; arrows in **f,g**). **(h)** Cells electroporated with EGFP-C1 developed as HCs (asterisk) and SCs (arrowheads). **(G,H)** Neurog1-AQ biased electroporated cells towards SC fate. Bars represent the number of cells counted in two consecutive frames of electroporated macula sacularis, from three independent embryos. Cell types were identified with the markers described above. In EGFP-C1 electroporation **(G)**, the fraction of electroporated cells (dark area) is similar for both HCs and SCs. However, after Neurog1-AQ electroporation **(H)**, very few electroporated cells became HCs (*n* = 3).

We analyzed first whether Neurog1 requires to bind the enhancer by testing the effects of a DNA-binding deficient Neurog1 (Neurog1-AQ, Sun et al., 2001) on the 3’Atoh1-enh. The experiments show that Neurog1-AQ was able to repress Atoh1, both *in ovo* and in P19 cells (Figures 5A,B). Neurog1-AQ also blocked Atoh1 autoactivation in 4xEnh B or 4xCAN constructs (Figures 5C,D) and, as expected, it did not activate the 4xEboxA (Figure 5E). This suggests that Neurog1 does not interfere with Atoh1 autoactivation by competing with Atoh1 for DNA binding or by the transcriptional activation of a repressor factor.

We also tested the ability of the DNA-binding deficient Neurog1 to inhibit HC formation in the embryo. Otic vesicles were electroporated at the prosensory stage (E3.5) and analyzed 3 days later, at the stage of HC differentiation (Figure 5). Electroporation of Neurog1 resulted in a massive neuronal commitment (Figure 5Fa) electroprated cells in non-neural domains were also forced to become neurons (Figure 5Fb). No electroporated cells were observed in the sensory epithelium after 3-days and HC patterning was not substantially affected (Figure 5Fc). Contrarily, electroporation of Neurog1-AQ caused a strong blockade of neuronal formation (Figure 5Fd), suggesting that it acts as a dominant-negative for Neurog1. In addition, Neurog1-AQ caused a strong bias towards the SC fate during HC formation. Neurog1-AQ electroporated cells remained in the epithelium and expressed Sox2 (Figures 5Fe–h). For EGFP electroporation, the proportion of HCs and SCs that were electroporated was similar (33% and 28%, respectively, Figure 5G). However, Neurog1-AQ (Figure 5H) reduced the fraction of electroporated HCs by about four-fold (from 33% to 8%, compare middle bars in Figures 5G,H), suggesting that Neurog1-AQ biased progenitors away from HC fate. Together, the results indicate that binding to DNA is dispensable for the repressor effect of Neurog1 on Atoh1 and HC formation.

**Figure 6:**
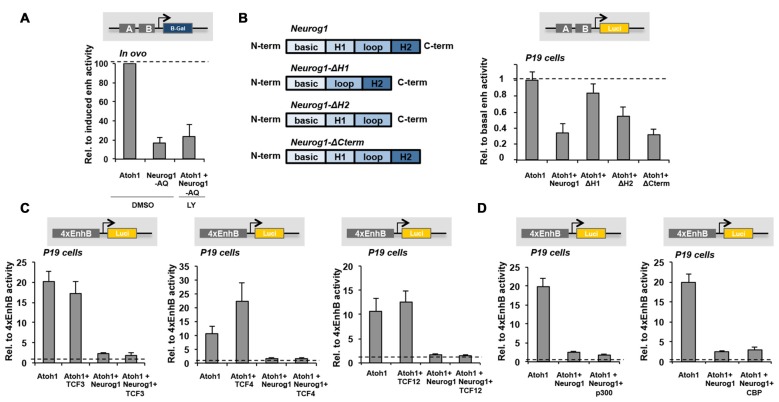
Neurog1 Helix-1 dimerization domain is essential for Atoh1 repression. **(A)** Neurog1 does not require Notch signaling for Atoh1 repression. E2 Otic vesicles were electroporated with the 3’Atoh1-enh w/o Neurog1-AQ. After 6 h, they were dissected and incubated with control or LY411575 o/n. The bars represent β-gal activity of the 3’Atoh1-enh relative to Atoh1 co-electroporation (*n* = 3). **(B)** Helix-1 is required for Neurog1 repression of the 3’Atoh1-enh. Left: diagram of the different constructs tested. Right: reporter activity in P19 cells shown in the conditions indicated in abscissa. Deletion of Helix-1 and to a less extent Helix-2 hampered the repression of Atoh1 autoactivation induced by Neurog1, which was unaltered by the C-term deletion (*n* = 4). **(C)** Overexpression of TCF3 (E47), TCF4 (E2.2) or TCF12 (HEB) E-proteins were unable to prevent repression of 4xEnhB by Neurog1 (*n* = 3–4). **(D)** Neither p300 nor CBP overexpression were able to prevent Neurog1 repression of 4xEnhB reporter activity in P19 cells (*n* = 3–14). H1: Helix-1, H2: Helix-2, N-term: N-terminal, C-term: C-terminal.

Given that the regions flanking the E-box A are typical binding sites for Notch-dependent bHLH repressors like Hes/Hey factors (Tateya et al., 2011; Du et al., 2013; Petrovic et al., 2014) and that Neurog1 requires these binding sites for its repressor function, we explored the effects of blocking Notch. Embryos were electroporated at E2 with the 3’Atoh1-enh and Atoh1 alone or combined with Neurog1-AQ. After 6 h otic vesicles were dissected, and incubated with or without the Notch inhibitor LY411575 for another 16 h. As shown, Neurog1-AQ repression was similar in both cases (Figure 6A), suggesting Notch signaling is not necessary for the repressor effect of Neurog1.

### Neurog1 Requires the Helix-1 Dimerization Domain for Atoh1 Repression

To gain insight into the mechanism of action of Neurog1, we analyzed the functionality of different modified Neurog1 constructs carrying selective deletions of the Helix-1, Helix-2 or the C-terminal domains. Deletion of Helix-1 restored almost completely Atoh1 autoactivation, and Helix-2 only partially (Figure 6B). The deletion of the C-terminal domain, which contains phosphorylation sites required for their activity and stability (Cundiff et al., 2009; Hardwick and Philpott, 2015), did not affect the repressor effect of Neurog1 (Figure 6B). Therefore, Helix-1 and Helix-2, which are dimerization domains, are crucial for the repressor function of Neurog1.

Atoh1 and Neurog1 bind to DNA by forming heterodimers with type I bHLH proteins named E-proteins. Examples are TCF3 (E47), TCF4 (E2.2) and TCF12 (HEB), which are broadly expressed during development (Murre et al., 1994; Kee, 2009). We tested the ability of these E-proteins to rescue Atoh1 repression by Neurog1, and the results showed that none of them was able to revert the effect of Neurog1 (Figure 6C). Neither p300/CBP nor Smad1 transcriptional coactivator components (Sun et al., 2001) were able to prevent the repression by Neurog1 (Figure 6D and data not shown). Therefore, Neurog1 does not seem to act by sequestering necessary transcriptional co-factors.

### Neurog1 Does Not Interact with Atoh1 Protein

**Figure 7:**
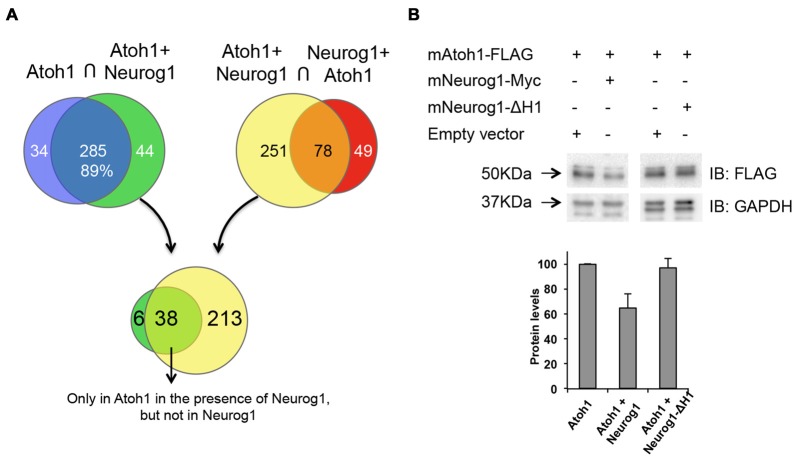
Neurog1 reduces Atoh1 protein levels. **(A)** Atoh1 interactome of P19 cells in the presence or absence of Neurog1 (see “Materials and Methods” section). Diagrams illustrate the number of identified proteins pulled down in the conditions indicated above. Some Atoh1 interacting proteins are lost (purple) or gained (green) in the presence of Neurog1, and some of them only interact either with Atoh1 (yellow) or with Neurog1 (red). The intersection between green and yellow (lower part) represents those proteins that interact only with Atoh1 in the presence of Neurog1 (Supplementary Table S6). **(B)** Neurog1 reduces Atoh1 protein levels. Western blot analysis of P19 cells transfected with Atoh1-FLAG in the presence or absence of Neurog1-Myc or Neurog1-∆H1. Bar diagram showing optical density values averaged from different experiments (*n* = 5 out of 7 for Neurog1 and *n* = 3 for Neurog1-∆H1). Values are relative to Atoh1-FLAG alone and normalized by GAPDH. IP, immunoprecipitation; IB, immunoblot.

We next questioned whether Neurog1 binds directly to Atoh1 forming a non-functional heterodimer. With this in mind we analyzed the Atoh1 interactome (IP Atoh1) and the Atoh1 interactome in the presence of Neurog1 (IP Atoh1-Neurog1). We also analyzed the interactome of Neurog1 in the presence of Atoh1 (IP Neurog1-Atoh1). This procedure provides also information on other possible proteins that interact with Atoh1. P19 cells were transfected with Atoh1-FLAG in the presence or absence of Neurog1-myc. Atoh1 was immunoprecipitated with an anti-FLAG antibody and the bound proteins identified by mass spectrometry. Neurog1 targeted several types of genes including genes that control regulation of transcription, signal transduction and cytoskeleton rearrangement, but with relative paucity of transcription factors (Supplementary Figure S3, see also Seo et al., 2007). TCF4 (E2.2) and TCF12 (HEB) were present in both immunoprecipitations (IP Atoh1 and IP Atoh1-Neurog1, Supplementary Table S3), indicating that E-proteins actually bind to Atoh1 regardless of the presence of Neurog1. Comparison of Atoh1 and Atoh1-Neurog1 interactomes, using SAINTexpress to score protein interactions, showed 89% overlap, indicating that a large fraction of proteins bind to Atoh1 regardless of the presence of Neurog1 (Figure 7A and Supplementary Table S4). The profile of the molecular function of both interactomes was very similar as well (Supplementary Figure S3).

Mass-Spectroscopy data showed that Neurog1 and Atoh1 did not co-immunoprecipitate, suggesting that they do not interact directly. Neither Neurog1 was pulled down by Atoh1 immunoprecipitation (Supplementary Tables S3, S5), nor Atoh1 was recovered after Neurog1 immunoprecipitation (IP Neurog1-Atoh1, Supplementary Tables S3, S5). This indicates that Neurog1 does not form non-functional heterodimers with Atoh1. Besides, the analysis of proteins that may interact with Atoh1 as activators gave no potential candidates for putative co-activators are displaced by Neurog1 (Supplementary Tables S4, S5).

### Neurog1 Reduces Atoh1 Protein Levels

**Figure 8:**
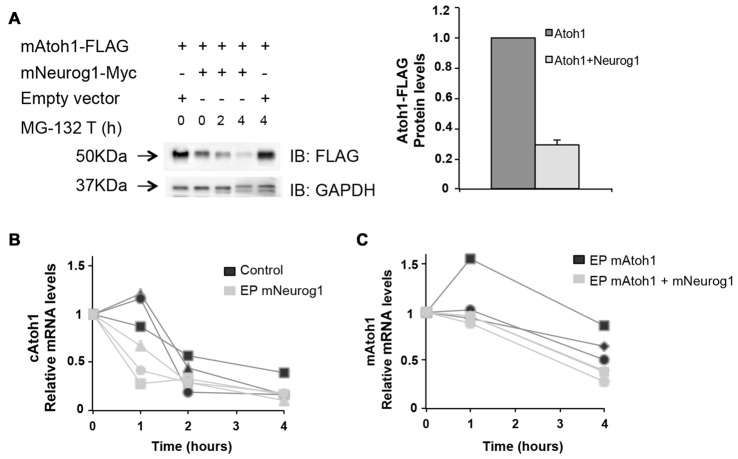
Neurog1 accelerates Atoh1 mRNA degradation. **(A)** Left: Western blot of mAtoh1-FLAG from electroporated chicken otic vesicles (lane 1). Neurog1 reduced Atoh1 protein levels (lane 2) but this reduction was not affected by the inhibition of proteosome degradation by MG-132 (lane 3-4). Right panel: Quantification of Atoh1-FLAG protein levels w/o Neurog1-Myc (lanes 1-2, *n* = 3). **(B)** Endogenous cAtoh1 mRNA levels in the presence of Actinomycin D in control or mNeurog1-Myc electroporated otic vesicles. Three independent experiments per condition are represented (black = control; gray = electroporated with mNeurog1). **(C)** Exogenous mAtoh1 RNA levels in the presence of Actinomycin D in otic vesicles electroporated with mAtoh1-FLAG w/o mNeurog1-Myc. Three independent experiments (black = electroporated with mAtoh1; gray = electroporated with mAtoh1+mNeurog1). Different experiments are labeled with different symbols. Messenger RNA from three otic vesicles per time point was retro-transcribed in duplicates and PCR with each pair of primers in triplicate PCR reactions (see “Materials and Methods” section).

Since the repression of Atoh1 by Neurog1 was independent on transcription or direct protein:protein interaction, we explored whether Neurog1 regulates Atoh1 protein levels. We transfected Atoh1-FLAG alone or in combination with Neurog1 and measured the tagged protein by Western Blot. This discards the effects of Atoh1 autoactivation, because atoh1-FLAG protein is constitutively expressed. Atoh1 expression was reduced in the presence of Neurog1, the reduction in optical density being of 36% in P19 cells (Figure 7B, bar diagram) and of 70% in chicken otic vesicles (Figure 8A). Interestingly, and like with the activation of the 3’Atoh1 enhancer (Figure 6B), the Helix-1 domain was necessary for the effect (Figure 7B, bar diagram, right).

Reduction in Atoh1 protein levels may be caused by increased Atoh1 protein degradation or by reduction of Atoh1 synthesis, the later being in turn related to impaired ribosomal function or to the reduction in mRNA levels. In the analysis of the IP we sorted out some proteins that only interact with Atoh1 in the presence of Neurog1. They included the E3 ubiquitin-protein ligase TRIM56 and the S-phase kinase-associated protein 1 (SKP1), a component of the SCF (SKP1-CUL1-F-box protein) ubiquitin ligase complex (Figure 7A and Supplementary Table S5). TRIM56 and SKP1 bound Atoh1 in the presence of Neurog1, but not to Neurog1 (Figure 7A and Supplementary Tables S3, S5). Since E3 ubiquitin ligases mediate polyubiquitination of proteins and target them to degradation, they were potential candidates to mediate the repressor effect of Neurog1.

With this in mind, otic vesicles were electroporated with Atoh1 and Neurog1 and then explanted and incubated in the presence or absence of the proteosome inhibitor MG-132 for 2 or 4 h. The results show that the inhibition of the proteosome pathway did not recover Atoh1 protein levels after Neurog1 co-electroporation (Figure 8A). Indeed, Neurog1 continued to reduce Atoh1 levels in the presence of the inhibitor. This indicates that Neurog1 does not reduce Atoh1 protein levels by targeting Atoh1 to proteosome-dependent degradation. We therefore tested whether Atoh1 mRNA levels were affected by Neurog1. E3 chicken otic vesicles were electroporated with Neurog1-Myc, and endogenous Atoh1 mRNA measured after transcriptional inhibition by Actinomycin D. Neurog1 accelerated the decay of endogenous Atoh1 mRNA after transcriptional blockade (Figure 8B). Moreover, Neurog1 was able to accelerate the degradation of overexpressed mouse Atoh1 in otic vesicles. Co-electroporation of mouse Atoh1-FLAG in the presence or absence of Neurog1-Myc showed exogenous mAtoh1 RNA was degraded faster in the presence of Neurog1 (Figure 8C).

In summary, the results suggest that the mechanism that accounts for the reduced levels of Atoh1 protein in the presence of Neurog1 is post-transcriptional. It does not seem to affect proteosome-dependent degradation, the experiments pointing to the regulation of mRNA stability as one potential mechanism.

## Discussion

### Neurons vs. Hair Cells

One central question in development is to understand how different cell types are generated at specific times and locations throughout embryonic life. The vertebrate inner ear is a simple example in which the three major cell types of its functional unit, HCs, SCs and neurons, arise with a precise timing and a specific spatial arrangement during development. The aim of the present work was to understand the molecular nature of cell fate decisions during inner ear development. In particular, we focused on the decision between neurogenesis and sensory development. The experiments reported here show that Neurog1 prevents the autoregulatory loop that drives Atoh1 expression and HC formation. The mechanism is cell autonomous and does not depend on Notch signaling. Further, the repression by Neurog1 is not due to its direct interaction with 3’Atoh-enhancer neither with Atoh1 protein, but by protein-protein interactions that result in the modification of Atoh1 mRNA stability and the decrease of Atoh1 protein levels. In other words, Neurog1 prevents Atoh1 transcription by lowering the levels of the major Atoh1 activator, which is Atoh1 itself. This explains why Neurog1 requires the integrity of the 3’ Atoh1-enh—the site of Atoh1 autoregulation—to exert its function, but not to bind DNA. The work provides a molecular explanation for the dominant effect of neurogenesis against sensorigenesis, and a novel mechanism for the interaction among proneural factors.

### The Enhancer B Accounts for the Atoh1 Autoregulatory Loop

Jane Johnson’s lab identified two conserved enhancers, A and B, within the 21 Kb regulatory sequence flanking the Atoh1 coding region (Helms et al., 2000; Ebert et al., 2003). Atoh1 binds to an E-box A located in Enhancer B, which is required for the activity of the transgenic expression of that enhancer (Helms et al., 2000; Lai et al., 2011). This suggested that the positive Atoh1 autoregulatory loop is the main mechanism for adjusting Atoh1 levels during development. ATAC-seq analysis and reporter analysis reveal that the EnhB is active in the embryo and drives Atoh1 activation. Although, EnhA shows no activity on its own, it modifies that of EnhB, being perhaps important for the spatial restriction and for the level of activation of EnhB. This is interesting in connection to the prosensoy function of Sox2. This factor activates the 3’Atoh1-enh by binding to EnhA, but it does not bind to EnhA in isolation (Neves et al., 2012 and data not shown), suggesting that Sox2 requires the physical interaction between the two enhancers (Ahmed et al., 2012). Sox2 has been identified recently as a pioneering transcription factor able to bind nucleosomal DNA (Soufi et al., 2015). Therefore, although EnhA displays little accessibility *in vivo*, it may be instrumental for Sox2-induced commitment of neurosensory progenitors. This interaction may be related to the epigenetic status of Atoh1 locus which, during organ of Corti development, shows a bivalent mark by H3K27me3 and H3K4me3, prior to its upregulation (Stojanova et al., 2015). This is consistent with the idea that Sox2 poises the Atoh1 locus until Atoh1 is able to bind to its 3’Atoh1-enh.

### The Flanking Region of E-box A Selects the Binding Affinity of Atoh1

The major activator of the 3’Atoh1-enhancer is Atoh1 itself, which binds to the E-box A located in the CAN region of EnhB. We show here that the flanking regions are crucial for Atoh1 activation and for the repression by Neurog1. E-box C is a preferred binding site for Type VI bHLH factors such as the Notch downstream targets Hey/Hes (Murre et al., 1994; Iso et al., 2003). A recent work shows that Hes5 and Hey2 prevent Atoh1 expression by binding to the promoter region in several *in vitro* cell lines (Abdolazimi et al., 2016). Transcriptional activity of Atoh1 is severely impaired by the mutation of the last nucleotides of the AtEAM motif, which is in agreement with the results from other model systems (Powell et al., 2004; Klisch et al., 2011). Accordingly, transcription is impaired after mutation of the N-box, but only when the AtEAM motif is affected. This indicates that the two last nucleotides of the AtEAM motif are crucial for proper Atoh1 function and that, in fact, the overlapping N-box is required for Atoh1 autoactivation. It is possible that they select the specificity of the type II bHLH factor that can sit on this E-box A.

### The Mechanism of Atoh1 Repression by Neurog1

Neurog1 is a transcriptional activator that binds to class A E-boxes (CANNTG; Bertrand et al., 2002; Evsen et al., 2013) and activates the expression of target genes like NeuroD (Ma et al., 1996; Kim et al., 2001). NeuroD suppresses Atoh1 expression in auditory-vestibular neurons as indicated by the ectopic expression of Atoh1 after its conditional deletion (Jahan et al., 2010). However, given that Neurog1 is expressed homogeneously in the neurosensory epithelium, including HC precursors (Raft et al., 2007), it is likely that alternative mechanisms collaborate to prevent Atoh1 without driving neuronal differentiation. Prevention of HC formation dissociated from neurogenesis may reinforce/enhance the progenitor neurosensory state (Sun et al., 2001; Fritzsch et al., 2006). Hence, the ability of Neurog1 to repress Atoh1 through a DNA-binding independent mechanism may be particularly adapted to this situation.

The experiments suggest that the repression of Atoh1 by Neurog1 relies on its ability to interfere with Atoh1 autoactivation. Helix domains are important for the dimerization ability of bHLH proteins and make significant contributions to the affinity of HLH interactions (Goldfarb et al., 1996; Longo et al., 2008). Helix-1 is essential for heterodimerization with other bHLH (Longo et al., 2008), but immunoprecipitations indicated that Neurog1 and Atoh1 do not bind directly. We tested whether co-factors like TCF3 (E47), TCF4 (E2.2) and TCF12 (HEB; Zhang et al., 2006; Flora et al., 2007) were sequestered by Neurog1, but none of them were able to prevent the repression by Neurog1 *in vitro*. This was also the case for other transcriptional co-activators like p300 or CBP, which are indeed sequestered by Neurog1 in neural stem cells (Sun et al., 2001). Sal1 protein, which prevents differentiation in other tissues (Basta et al., 2014) was among the proteins bound to Atoh1 in the presence of Neurog1, but there is no information on its function during ear development.

One general mechanism for the regulation of long-term protein interactions is the control of protein levels. The decrease of Atoh1 protein levels induced by Neurog1 was abolished when Neurog1 lacked its Helix-1 domain, suggesting a common link for both the loss of Atoh1 protein and the repressor effect on the 3’Atoh1-enh. The reduction in Atoh1 protein levels, in turn, may be caused by at least these three mechanisms, which are not mutually exclusive: first, the decrease of Atoh1 mRNA, second, the reduction of Atoh1 translation rate and finally, the increase of Atoh1 degradation. Our results suggest that the regulation of Atoh1 mRNA stability by Neurog1 is a potential mechanism for reducing Atoh1 protein levels. How this occurs requires further study and we are far from understanding how Neurog1 may target Atoh1 mRNA for degradation. Neither we know whether Neurog1 also affects ribosomal translation. Neurog1 needs the integrity of its helix-1 heterodimerization domain for reducing both Atoh1 protein and Atoh1 enhancer activity, but we have no hint on the nature of the partners that may connect Neurog1 to mRNA regulation and Atoh1 translation. Micro RNAs (miRNAs), which bind to target messenger RNA transcripts and reduce translation, are interesting candidates to be involved in such a network (Groves et al., 2013), however, we have no evidence on how they could be related to Neurog1 expression.

## Author Contributions

Experimental work was carried out by HG, GA and FG; data analysis by HG, GA, JJT and FG; manuscript writing by HG, GA and FG; funding and project management by FG.

## Conflict of Interest Statement

The authors declare that the research was conducted in the absence of any commercial or financial relationships that could be construed as a potential conflict of interest.
