# Supplementary material for: The Repression of Atoh1 by Neurogenin1 during Inner Ear Development
Source: Front Mol Neurosci. 2017 Oct 20;10:321. doi: 10.3389/fnmol.2017.00321 (PMC5655970; doi:10.3389/fnmol.2017.00321)
Supplement: Supplementary file 2 [file Table_2.docx]

**SUPPLEMENTARY TABLE 2**

**Table S2. Expression vectors.** (+) Vectors used in ovo and (*) vectors used in P19 cells.
