# Supplementary material for: The Repression of Atoh1 by Neurogenin1 during Inner Ear Development
Source: Front Mol Neurosci. 2017 Oct 20;10:321. doi: 10.3389/fnmol.2017.00321 (PMC5655970; doi:10.3389/fnmol.2017.00321)
Supplement: Supplementary file 7 [file Data_Sheet_1.docx]

**SUPPLEMENTARY FIGURES**


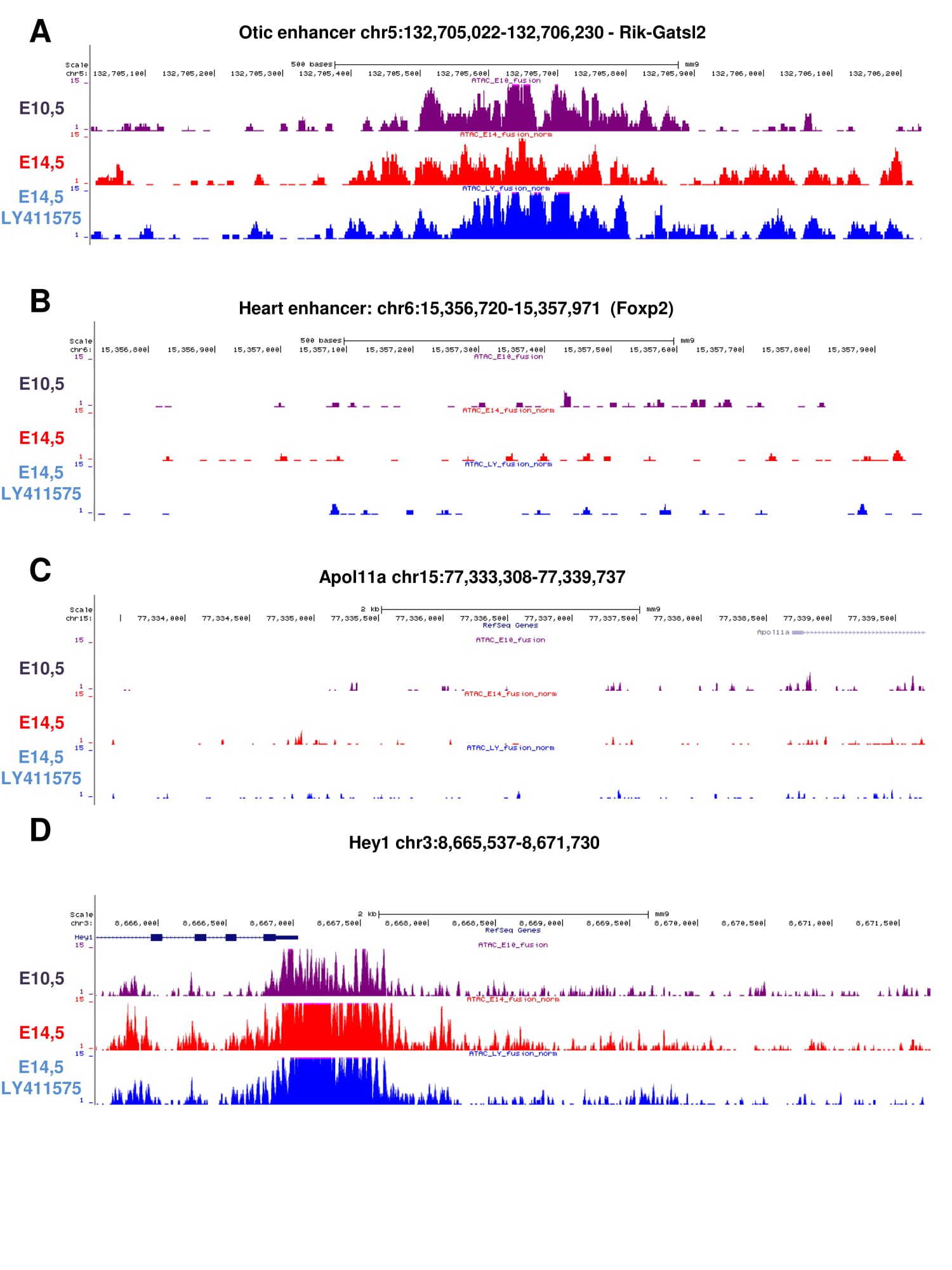


**Fig. S1. ATAC-Seq data validation.** **(A)** ATAC-seq profile of the otic enhancer Rik-Gatsl2 annotated in the VISTA Enhancer Browser. The enhancer was accessible in all three conditions. **(B)** The region of a heart enhancer for Foxp2 gene was not accessible in the otic samples tested. **(C)** The promoter of Apol11a gene, which is not expressed in the ear, was not accessible. **(D)** The promoter of Hey1 gene, was accessible in the three conditions tested.

For further confirmation we have also compared our ATAC data with already tested enhancers available in Vista Browser (Visel et al. Ultraconservation identifies a small subset of extremely constrained developmental enhancers. Nature Genet. 2008). We downloaded data from Vista Browser [https://enhancer.lbl.gov](https://enhancer.lbl.gov/) corresponding to 15 enhancers that are active only in the inner ear and 327 enhancers common to neural tissues (cranial nerve, dorsal root ganglion, ear, eye, forebrain, hindbrain (rhombencephalon), midbrain (mesencephalon), neural tube), and compared them to our ATAC peaks. By this procedure we identified 12 otic enhancers (80%) and 235 “neural” peaks (72%) that are recovered in our ATAC sequences. If instead we test random regions of the same size of the actual enhancers, then only 1 out of 15 (6%) and 34 out of 327 (10%) are present in our ATAC data.


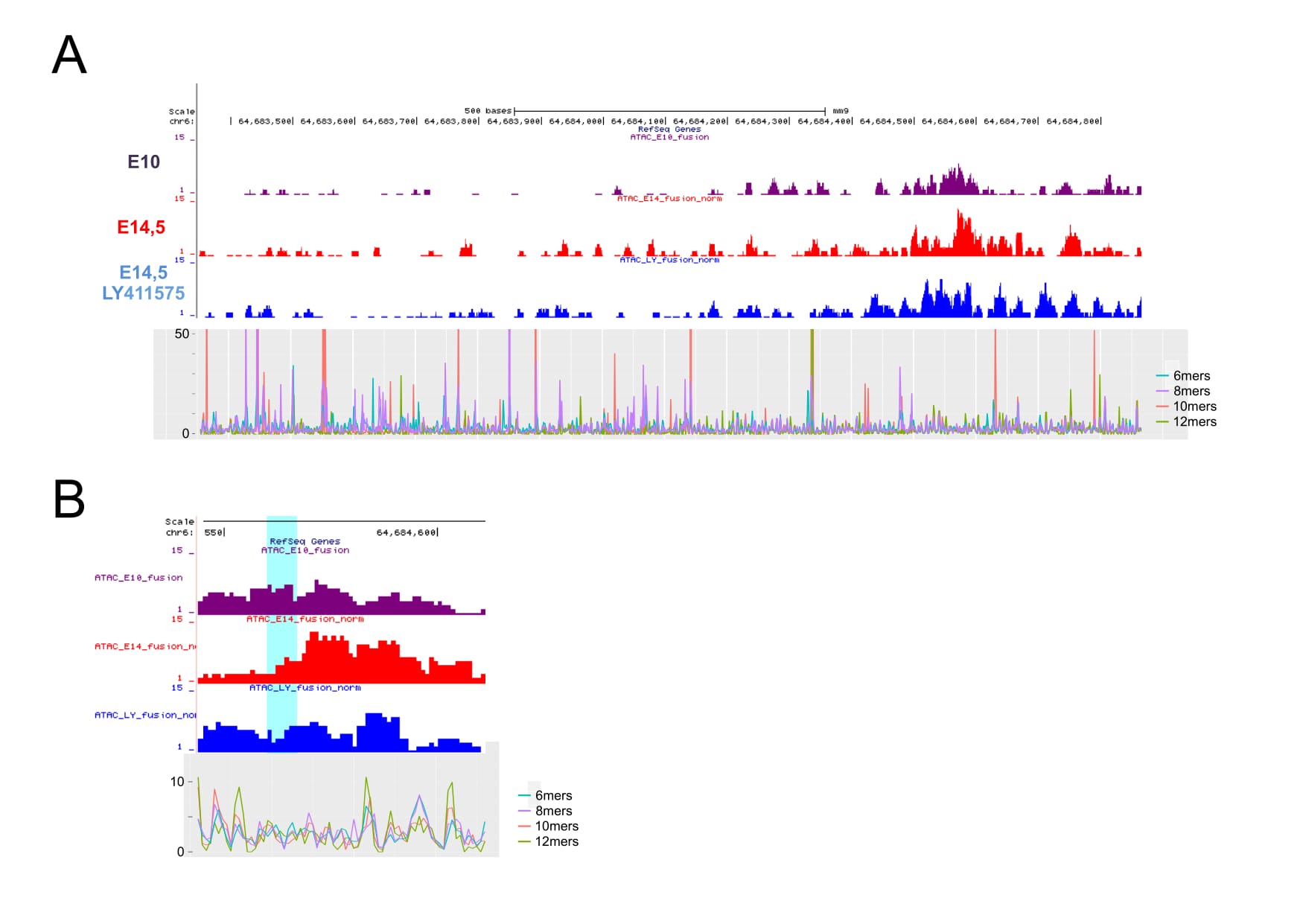
**Fig. S2. Transposase bias analysis** from ATAC peaks of all samples (almost 200,000 peaks). We calculated the number of Tn5 cuts in each kmer (fragment of k bp). This value was divided by the number of times that kmer appears in all the peaks (A). This results in an average number of cuts in that kmer. If the Tn5 has a preference for a particular kmer, this average number of cuts will come out much higher. Since *a priori* we do not know if the Tn5 prefers kmers of a certain size, we have tested with k = 6, 8, 10 and 12. This does not show that the Tn5 has no bias, but that if it exists, the bias does not explain the signal we see. Once we calculated the average number of cuts for each kmer, we went to the region interest and represented that number for each kmer of that region (B). The results show that these values and the ATAC signal are not correlated.


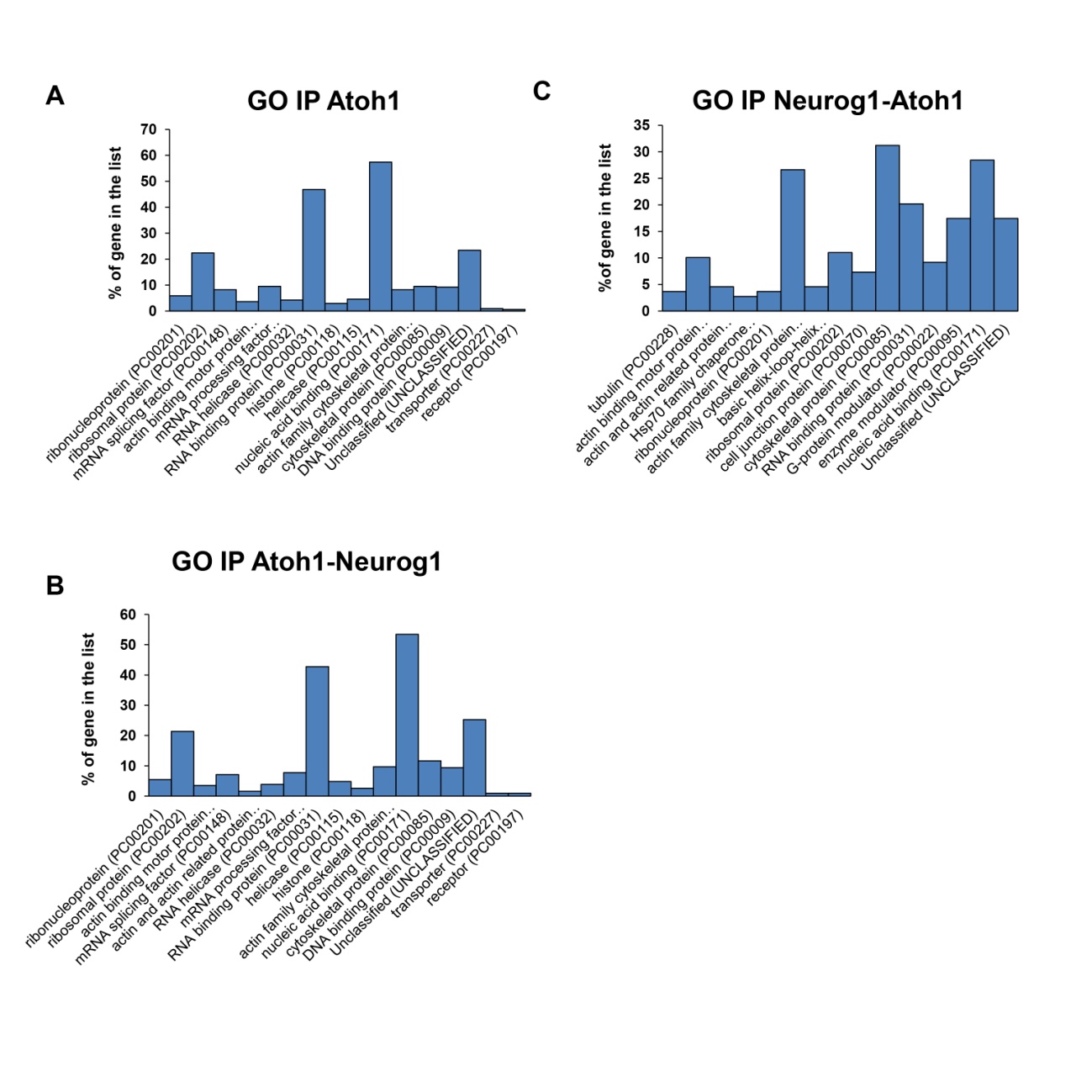


**Fig. S3. Analysis of the immunoprecipitated interactors**. Each list of immunoprecipitated proteins were classified by protein class using gene ontology with the data set PANTER class**. (A)** Classification of the IP Atoh1 **(B)** IP Atoh1-Neurog1 and **C)** IP Neurog1-Atoh1. Detailed information for IP-mass spec: Beads were washed three times with 500μl of 0.2M NH4HCO3, resuspended in 60μl of 6M urea 0.2M NH4HCO3, reduced with dithiotrhreitol (10μl DTT 10mM, 37ºC, 60min), alkylated with iodoacetamide (10μl IAM 20mM, 25ºC, 30min), diluted up to 1M urea with 0.2M NH4HCO3 and digested overnight with trypsin (1μg, 37ºC). Samples are desalted using C18 stape tips (UltraMicroSpin Column, SUM SS18V, The Nest group,Inc., MA). 45% of the peptide mixture was analyzed using a LTQ-Orbitrap Velos Pro mass spectrometer (Thermo Fisher Scientific, San Jose, CA) coupled to a nano-LC (Proxeon, Odense, Denmark) equipped with a reversed-phase chromatography 2-cm C18 pre-column (Acclaim PepMap-100, Thermo; 100µm i.d., 5µm), and a reversed-phase chromatography 25cm column with an inner diameter of 75 μm, packed with 1.9 μm C18 particles (Nikkyo Technos, Japan). Chromatographic gradients started at 7% buffer B with a flow rate of 300 nL/min and gradually increased to 35% buffer B in 60 min. After each analysis, the column was washed for 15min with 90% buffer B (Buffer A: 0.1% formic acid in water. Buffer B: 0.1% formic acid in acetonitrile). The mass spectrometer was operated in positive ionization mode with nanospray voltage set at 2.2 kV and source temperature at 300 °C. Ultramark 1621 for the FT mass analyzer was used for external calibration prior the analyses. The background polysiloxane ion signal at m/z 445.1200 was used as lock mass. The instrument was operated in data-dependent acquisition (DDA) mode, and full MS scans with 1 microscan at resolution of 60 000 were used over a mass range of m/z 350−2000 with detection in the Orbitrap. Auto gain control (AGC) was set to 1e6, dynamic exclusion was set at 60s, and the charge-state filter disqualifying singly charged peptides for fragmentation was activated. Following each survey scan, the 20 (CID) most intense ions with multiple charged ions above a threshold ion count of 5000 were selected for fragmentation at normalized collision energy of 35%. Fragment ion spectra produced via CID were acquired in the linear ion trap, AGC was set to 1e4 and isolation window of 2.0 m/z, activation time of 10 ms and maximum injection time of 100 ms were used. All data were acquired with Xcalibur software v2.2. Data Analysis Acquired data were analyzed using the Proteome Discoverer software suite (v1.4.1.14, Thermo Fisher Scientific), and the Mascot search engine (v2.5.1, Matrix Science (Perkins et al., 1999) was used for peptide identification. Data were searched against a Mus musculus protein database derived from SwissProt plus the most common contaminants (total of 17335 sequences). A precursor ion mass tolerance of 7 ppm at the MS1 level was used, and up to three missed cleavages for trypsin were allowed. The fragment ion mass tolerance was set to 0.5 Da. Oxidation of Methionine and N-terminal protein acetylation was defined as variable modification and carbamidomethylation of Cysteines was set as fixed modification. The identified peptides were filtered 5%FDR. SAINTexpress (Teo et al., 2014) was used to score protein interactions.
